# Functional Characterization of Endo- and Exo-Hydrolase Genes in Arabinan Degradation Gene Cluster of *Bifidobacterium longum* subsp. *suis*

**DOI:** 10.3390/ijms25063175

**Published:** 2024-03-09

**Authors:** Yewon Kang, Chang-Yun Choi, Jihun Kang, Ye-Rin Ju, Hye Bin Kim, Nam Soo Han, Tae-Jip Kim

**Affiliations:** 1 Division of Animal, Horticultural and Food Sciences, Graduate School of Chungbuk National University, Cheongju 28644, Republic of Korea; wid9485@naver.com (Y.K.); cychoi@aptech.biz (C.-Y.C.); kimcholl@naver.com (J.K.); juyelin@naver.com (Y.-R.J.); hyebin_21@naver.com (H.B.K.); namsoo@cbnu.ac.kr (N.S.H.); 2 Department of Food Science and Biotechnology, Chungbuk National University, Cheongju 28644, Republic of Korea

**Keywords:** *Bifidobacterium longum* subsp. *suis*, arabinan degradation gene cluster, endo-α-(1,5)-l-arabinanases (ABNs), exo-α-l-arabinofuranosidases (ABFs), hydrolytic modes of action

## Abstract

Bifidobacteria are probiotic microorganisms commonly found in the gastrointestinal tract, some of which are known to utilize linear arabino-oligosaccharides (AOS) as prebiotic carbohydrates. In general, the synergistic actions of exo-type α-l-arabinofuranosidases (ABFs) and endo-α-1,5-l-arabinanases (ABNs) are required for efficient arabinan degradation. In this study, the putative gene cluster for arabinan degradation was discovered in the genome of *Bifidobacterium longum* subsp. *suis*. It consists of a variety of genes encoding exo- and endo-hydrolases, sugar-binding proteins, ABC-binding cassettes, and transcriptional regulators. Among them, two endo-ABNs GH43 (BflsABN43A and BflsABN43B), two exo-ABFs GH43 (BflsABF43A and BflsABF43B), and an exo-ABF GH51 (BflsABF51) were predicted to be the key hydrolases for arabinan degradation. These hydrolase genes were functionally expressed in *Escherichia coli*, and their enzymatic properties were characterized. Their synergism in arabinan degradation has been proposed from the detailed modes of action. Extracellular endo-BflsABN43A hydrolyzes sugar beet and debranched arabinans into the short-chain branched and linear AOS. Intracellularly, AOS can be further degraded into l-arabinose via the cooperative actions of endo-BflsABN43B, exo-BflsABF43A with debranching activity, α-1,5-linkage-specific exo-BflsABF43B, and exo-BflsABF51 with dual activities. The resulting l-arabinose is expected to be metabolized into energy through the pentose phosphate pathway by three enzymes expressed from the *ara* operon of bifidobacteria. It is anticipated that uncovering arabinan utilization gene clusters and their detailed functions in the genomes of diverse microorganisms will facilitate the development of customized synbiotics.

## 1. Introduction

Bifidobacteria are anaerobic, non-motile, non-spore-forming, and Gram-positive microorganisms. They are the dominant populations in the human gastrointestinal tract, and are considered the representative probiotic microorganisms. The health-beneficial effects of bifidobacteria include alleviating lactose intolerance, irritable bowel disease, antibiotics-associated diarrhea, and inflammatory bowel disease [1]. When prebiotics are fermented by the gut microorganisms, health-beneficial compounds such as short-chain fatty acids (SCFAs) and metabolic energy are produced. Prebiotics provide health benefits such as immune function, metabolic health, anti-obesity, anti-allergy, and so on [2]. Nowadays, global interest in synbiotics, which focuses on the synergistic effects between probiotic microorganisms and prebiotic substances, continues to grow [3]. In addition to the hexose-based prebiotics such as fructo-oligosaccharides (FOS) and galacto-oligosaccharides (GOS), the novel health functionality and promising prebiotic effects of hemicellulose polymers and their oligosaccharides, composed of the pentoses l-arabinose and d-xylose, are receiving a lot of attention [4,5,6,7,8]. Arabinoxylans are complex hetero-polysaccharides consisting of a main chain of β-1,4-linked d-xyloses with side branches of l-arabinose attached via α-1,2 and/or α-1,3-bonds. These polymers are hydrolyzed into monosaccharides through the synergistic actions of various hydrolases [9,10,11,12]. Arabinans are homo-polysaccharides consisting of α-1,5-l-arabinofuranosyl backbone and α-1,2- and/or α-1,3-l-arabinofuranosyl branches. These hemicellulose polymers are enzymatically hydrolyzed into arabino-oligosaccharides (AOS) and l-arabinose [13]. l-arabinose is known to inhibit intestinal sucrase activity, decreasing sucrose absorption, which in turn lowers blood glucose levels and helps prevent obesity [14]. Accordingly, l-arabinose, AOS, and arabinans can be promising functional prebiotic candidates as the growth promoters for probiotic microorganisms such as bifidobacteria [4,6,8,15].

To utilize arabinan and its hydrolysates as prebiotic carbon sources, microorganisms possess corresponding gene clusters consisting of endo- and exo-acting arabinan-degrading enzyme genes, carbohydrate transporter genes, and a set of enzymes involved in l-arabinose metabolism. Among arabinan-degrading enzymes, exo-acting α-l-arabinofuranosidases (ABFs; EC 3.2.1.55) are involved in the degradation of arabinoxylans and arabinogalactan, as well as arabinans [16]. These enzymes belong to the Glycoside Hydrolase (GH) 2, 3, 43, 51, 54, and 62 families [http://www.cazy.org, accessed on 10 January 2024], which catalyze the hydrolysis of terminal non-reducing α-1,2-, α-1,3-, and/or α-1,5-arabinofuranoside residues from AOS and arabinans. Most ABFs have been found in various bacteria, such as *Cellvibrio* [17], *Streptomyces* [18], *Thermotoga* [19], *Lactobacillus* [20], *Bacillus* [21,22], *Caldicellulosiruptor* [23], *Geobacillus* [24], *Clostridium* [25], *Bifidobacterium* [26,27], and *Weissella* [28] species and so on, as well as from fungi and yeasts [29,30]. Meanwhile, endo-α-1,5-l-arabinanases (ABNs; EC 3.2.1.99) belong to the GH43 family and specifically hydrolyze arabinans into short-chain AOS. Compared with the intensive works on ABFs, only limited numbers of microbial ABNs have been studied from *Aspergillus* [31], *Cellvibrio* [32], *Penicillium* [33], *Bacillus* [34,35], *Caldicellulosiruptor* [36], *Geobacillus* [37], *Phanerochaete* [38], *Thermotoga* [39], *Rhizomucor* [40], *Caldanaerobius* [41], and *Lactobacillus* [42] species, and so on.

The microbial arabinan utilization system was first reported from *Bacillus subtilis* 168T^+^, which possesses two exo-ABFs belonging to the GH51 family (AbfA and Abf2) [21] and two endo-ABNs GH43 (AbnA and Abn2) [43,44]. Extracellular AbnA and Abn2 hydrolyze arabinans to produce AOS, whereas intracellular AbfA and Abf2 saccharify AOS into l-arabinose. Intracellularly, AbfA hydrolyzes both sugar beet (branched) arabinan and debranched (linear) arabinan, whereas Abf2 is active only on sugar beet arabinan, not on debranched arabinan. However, their specific hydrolytic modes of action have not been fully elucidated. Similar arabinan utilization systems have also been reported in *Hypocrea jecorina* (*Trichoderma reesei*) [45], *Corynebacterium glutamicum* [46], *Geobacillus stearothermophilus* [47], *Caldanaerobius polysaccharolyticus* [41], and *Lactobacillus crispatus* [42].

From bifidobacteria, gene functions have mainly been characterized for various exo-type hydrolases, including α-l-arabinopyranosidase [48], β-l-arabinofuranosidase [49], α-d-galactosidase [50], and β-d-galactosidase [51] as well as ABFs, while no endo-type bifidobacterial ABN has been reported yet. In the case of ABF enzymes, they can also play important roles as accessory enzymes, with l-arabinose-debranching activity, in utilizing heteropolysaccharides such as arabinoxylans and arabinogalactans as well as arabinans, while the ABN enzymes act specifically only on α-1,5-arabinofuranosidic linkages in arabinans. Therefore, ABNs are key players that can be uniquely found only in arabinan utilization systems. In this study, two open reading frames (ORFs) encoding putative endo-type ABNs were first discovered from the genome of *Bifidobacterium longum* subsp. *suis* ATCC 27533 [52], and the structure of the arabinan utilization gene cluster was predicted and analyzed. In addition, a total of five genes encoding endo- and exo-arabinan hydrolases were cloned and expressed in *Escherichia coli*, and their enzymatic properties were characterized in detail. Their hydrolytic modes of action in arabinan degradation can provide us scientific insights into the enzymatic prebiotic utilization systems in probiotic bifidobacteria.

## 2. Results

### 2.1. Gene Cluster Analysis for Arabinan Utilization in Bf. longum subsp. suis

**Figure 1:**
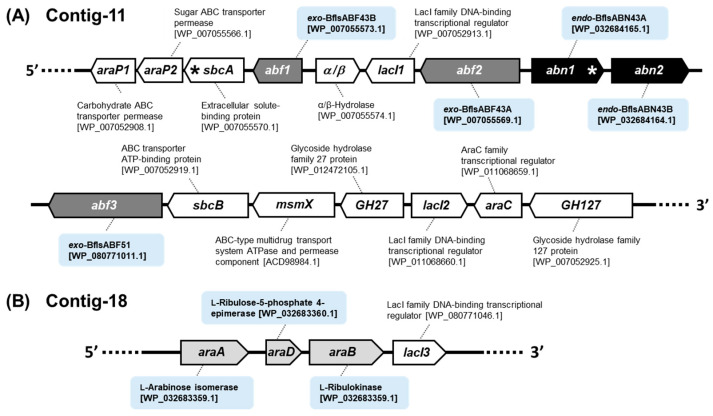
Arabinan utilization gene cluster predicted from (**A**) contig-11 and (**B**) contig-18 in the genome of *Bifidobacterium longum* subsp. *suis* ATCC 27533 (GenBank ID: GCA_000771285.1). The genes marked with an asterisk were expected to possess a signal sequence for secretion predicted by SignalP 5.0. Genes encoding endo-ABNs (black), exo-ABFs (dark gray), and *ara* operon (light gray) were functionally characterized in this study.

Despite the unique and crucial role of extracellular endo-ABNs in depolymerizing arabinan homopolymers into smaller AOS for cellular uptake, there have been no reports on the characteristics of these hydrolases derived from bifidobacteria to date. The BLAST amino acid sequence homology search revealed that two genes encoding putative ABNs were found in the genome of the *Bifidobacterium longum* subsp. *suis* type strain ATCC 27533 (= DSM 20211 = KCTC 3229; hereafter, *Bf. longum suis*). This Gram-positive and anaerobic bacterium with high (G+C)% is known to be isolated from pig intestines [52], and its genome sequence was partially determined into total 129 contigs (GenBank ID: GCA_000771285.1). Genome analysis suggested that contig-11 includes the probable arabinan degradation gene cluster containing total five core genes encoding arabinan-degrading enzymes: two endo-ABNs GH43 (BflsABN43A and BflsABN43B), two exo-ABFs GH43 (BflsABF43A and BflsABF43B), and an exo-ABF GH51 (BflsABF51). Within this gene cluster, two more genes encoding the functionally unknown hydrolases GH27 and GH127 (putative α-galactosidase and β-l-arabinofuranosidase, respectively), *lacI* transcriptional regulator, and sugar ABC transporter protein genes were also included. Meanwhile, the *ara* operon, consisting of *araA* (l-arabinose isomerase), *araD* (l-ribulose-5-phosphate 4-epimerase), and *araB* (l-ribulokinase) genes, was found in the contig-18. The secretion of each enzyme into the extracellular space was theoretically predicted using SignalP 5.0. Among five putative arabinan hydrolases, only BflsABN43A was predicted to be secreted extracellularly, as it possesses a signal peptide sequence of 35 amino acids at its N-terminus. In contrast, the other four hydrolases are expected to be responsible for degradative functions within the cell of *Bf. longum suis* (Figure 1).

**Figure 2:**
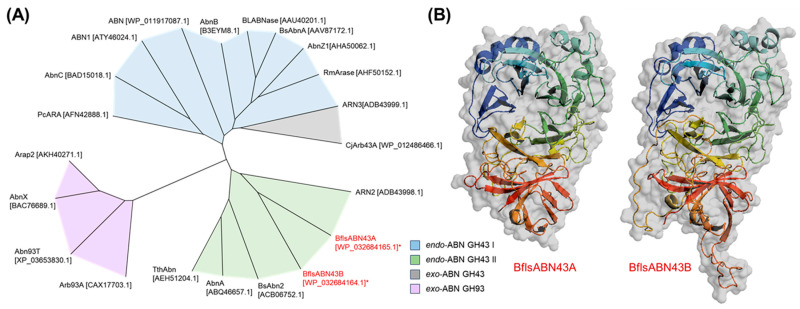
(**A**) Phylogenetic relationship among various microbial endo-ABNs and (**B**) 3D structure prediction of BflsABNs. The abbreviations for ABNs include BflsABN43A and BflsABN43B, ABNs from *Bifidobacterium longum* subsp. *suis* ATCC 27533 (with an asterisk); PcARA, from *Phanerochaete chrysosporium*; AbnC, from *Penicillium chrysogenum* 31B; ABN1, from *Penicillium purpurogenum*; ABN, from *Caldicellulosiruptor saccharolyticus*; AbnB, from *Geobacillus stearothermophilus* T-6; BsAbnA, from *Bacillus subtilis* 168T^+^; BLABNase, from *Bacillus licheniformis* DSM13: BsAbnA, from *Bacillus subtilis* 168T^+^; AbnZ1, from *Paenibacillus polymyxa*; RmArase, from *Rhizomucor miehei*; CjArb43A, from *Cellvibrio japonicus*; ARN2 and ARN3, from bovine ruminal metagenome; BsAbn2, from *Bacillus subtilis* 168T^+^; AbnA, from *Thermotoga petrophila* RKU-1; TthAbn, from *Thermotoga thermarum* DSM 5069; Arb93A, from *Fusarium graminearum*; Abn93T, from *Thermothielavioides terrestris*; AbnX, from *Penicillium chrysogenum* 31B; Arap2, from *Penicillium purpurogenum*. Three-dimensional structure models consisting of cartoon and surface predicted by AlphaFold2 were visualized using PyMOL, colored in a rainbow spectrum from the N-terminus (blue) to the C-terminus (red).

Due to their limited distribution in nature, only a few ABNs have been reported from different genus of *Bacillus* [34,35,43,44], *Aspergillus* [31], and *Lactobacillus* [42]. Two endo-type hydrolases, an extracellular BflsABN43A and an intracellular ABN43B, consist of 531 and 581 amino acids, respectively. They share a high amino acid sequence identity of 41% with each other, while they showed relatively low sequence identities of less than 30% with the other known ABNs GH43. BflsABN43 enzymes share the highest sequence identities (23~29%) with Abn2 GH43 from *Bacillus subtilis* [44], which can hydrolyze both sugar beet arabinan and debranched arabinan. On the contrary, BflsABN43 showed significantly lower sequence identities (9~13%) with *B. subtilis* AbnA GH43 [43] and *B. licheniformis* ABN GH43 [34], which are specific for only debranched and linear arabinans. Based on the phylogenetic relations derived from amino acid sequences, in this study, endo-acting ABN GH43 enzymes were categorized into two groups: I and II. In general, group I ABNs are expected to hydrolyze mainly linear substrates, whereas group II can attack both branched and linear substrates. Both BflsABN43A and ABN43B are assumed to be members of endo-ABN GH43 group II (Figure 2A).

From the tertiary structural aspect, microbial group I endo-ABNs GH43 were shown to possess a core catalytic domain with a five-bladed β-propeller architecture [32,35,37]. Meanwhile, group II ABNs contain an additional C-terminal jelly-roll domain as well as a core catalytic domain [39,53]. Based on amino acid sequences, the three-dimensional structures of BflsABNs GH43 were predicted by using the AlphaFold2 structure modeling server [https://alphafold.ebi.ac.uk]. Both BflsABNs GH43 are expected to share a catalytic five-bladed β-propeller fold and a C-terminal jelly-roll domain with ABNs GH43 group II, such as BsAbn2 from *B. subtilis* [53] (Figure 2B). Despite the relatively low similarity of less than 30% of the primary structure, BflsABNs GH43 share closely similar tertiary structure and catalytic amino acid residues with other known endo-ABNs GH43 in group II. Except for a few extra-loop conformations, the overall tertiary structure of BflsABN43B was almost similar to BflsABN43A.

**Figure 3:**
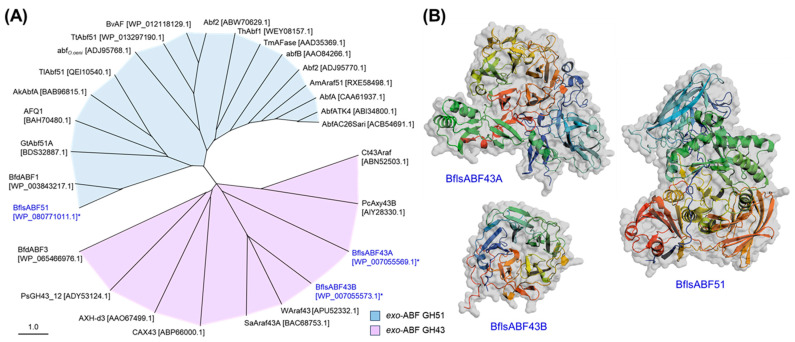
(**A**) Phylogenetic relationship among various microbial exo-ABFs and (**B**) 3D structure prediction of BflsABFs. The abbreviations for ABFs: BflsABF51, BflsABF43A, and BflsABF43B, ABFs from *Bifidobacterium longum* subsp. *suis* ATCC 27533 (with an asterisk); BfdABF1 and BfdABF3, from *Bifidobacterium dentium* ATCC 27679; GtAbf51A, from *Gloeophyllum trabeum*; AFQ1, from *Penicillium chrysogenum* 31B; AkAbfA, from *Aspergillus kawachii* IFO 4308; TlAbf51, from *Talaromyces leycettanus* JCM 12802; abf_O.oeni_, from *Oenococcus oeni* ATCC BAA-1163; TtAbf51, from *Thermoanaerobacterium thermosaccharolyticum* DSM 571; BvAF, from *Bacillus velezensis* FZB42; AbfA and Abf2, from *Bacillus subtilis* 168; ThAbf1, from *Trametes hirsute*; TmAFase, from *Thermotoga maritima* MSB8; abfB, from *Bifidobacterium longum* B667; Abf2, from *Lactobacillus brevis* DSM 20054; AmAraf51, from *Acetivibrio mesophilus*; AbfATK4, from *Geobacillus caldoxylolyticus* TK4; AbfAC26Sari, from *Anoxybacillus kestanbolensis* AC26Sari; Ct43Araf, from *Clostridium thermocellum*; PcAxy43B, from *Paenibacillus curdlanolyticus* B-6; WAraf43, from *Weissella* sp. 142; SaAraf43A, from *Streptomyces avermitilis* NBRC14893; CAX43, from *Caldicellulosiruptor saccharolyticus* DSM 8903; AXH-d3, from *Bifidobacterium adolescentis* DSM 20083; PsGH43, from *Pseudopedobacter saltans*. Three-dimensional structure models consisting of cartoon and surface predicted by AlphaFold2 were visualized using PyMOL, colored in a rainbow spectrum from the N-terminus (blue) to the C-terminus (red).

Three intracellular exo-type hydrolases, BflsABF43A, ABF43B, and ABF51, encode 710, 344, and 823 amino acids, respectively. BflsABF43A shares much low-sequence identities, less than 13%, with BflsABF43B and the other known ABNs GH43. On the other hand, BflsABF43B showed many high-sequence identities of 48% and 32% with WAraf43 and SaAraf43A, exo-α-1,5-l-arabinofuranosidases GH43, from *Weissella* [28] and *Streptomyces avermitilis* [54], respectively. These ABFs GH43 share a catalytic five-bladed β-propeller fold with each other, and specifically hydrolyze α-1,5-linked small AOS to l-arabinose. Meanwhile, BflsABF51 showed the amino acid sequence identity of 12% and 7% with BflsABF43A and BflsABF43B, respectively. It showed the highest amino acid sequence identities of 67% with BfdABF1, an exo-ABF GH51 highly active on small arabinoxylo-oligosaccharides (AXOS), originating from *Bifidobacterium dentium* [26], while it showed very low sequence identity, less than 15%, with the other known microbial ABFs GH51 such as GtAbf51A (Figure 3A).

Due to their versatile functions in hemicellulose degradation and wide distribution in nature, various microbial ABFs, belonging to GH2, 3, 43, 51, 54, and 62 families, have been reported to date. The previous structural studies suggested that ABFs GH43 and ABF51 possess a five-bladed β-propeller fold and a catalytic (β/α)_8_-barrel fold with C-terminal jelly-roll domain [19,28,54,55,56], respectively. The structure of ABF GH54, consisting of β-sandwich folded catalytic and arabinose-binding domains, was also reported [57]. The structural models of BflsABFs were predicted and compared with each other (Figure 3B). Although both BflsABFs GH43 share a five-bladed β-propeller catalytic fold, BflsABF43A possesses extra-N-domains with complexed β/α-folds. BflsABF51 has a highly complex structure consisting of an N-terminal jelly-roll domain, a typical (β/α)_8_-barrel core catalytic domain, and two C-terminal jelly-roll domains.

### 2.2. Gene Expression and Enzyme Purification of Arabinan Hydrolases

**Figure 4:**
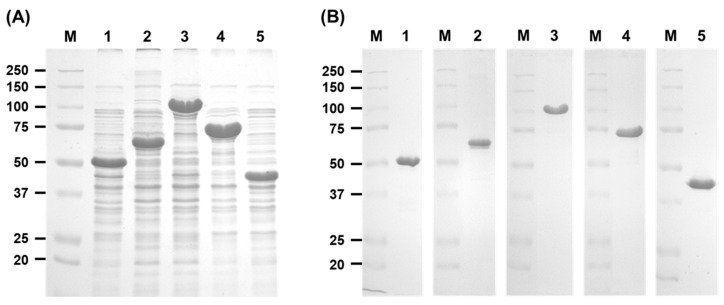
SDS-PAGE analysis for (**A**) gene expression and (**B**) enzyme purification of arabinan hydrolases from *Bifidobacterium longum* subsp. *suis* expressed in recombinant *E. coli*. Lane M, protein size marker; 1, BflsABN43A; 2, BflsABN43B; 3, BflsABF51; 4, BflsABF43A; 5, BflsABF43B.

To investigate the functionality of the hypothetical arabinan hydrolases from *Bf. longum suis*, the corresponding enzyme genes were cloned and expressed in *E. coli*, followed by a comparative analysis of their enzymatic characteristics. The genes encoding BflsABN43A (GenBank ID: WP_032684165.1, 531 amino acids, 56 kDa), BflsABF43A (WP_032684165.1, 710 amino acids, 77 kDa), and BflsABF43B (WP_007055573.1, 344 amino acids, 38 kDa) were inserted into the pHCXHD expression vector and constitutively expressed in *E. coli* MC1061. Additionally, the genes for BflsABN43B (WP_032684164.1, 581 amino acids, 65 kDa) and BflsABF51 (WP_080771011.1, 823 amino acids, 90 kDa) were cloned into the pET-21a(+) vector and induced for expression in *E. coli* BL21(DE3) using IPTG inducer. Consequently, all arabinan hydrolase genes, fused with a C-terminal six-histidines tag, were overexpressed and purified to a homogeneity through Ni-NTA affinity chromatography (Figure 4).

### 2.3. Enzymatic Characterization of Arabinan Hydrolases

**Figure 5:**
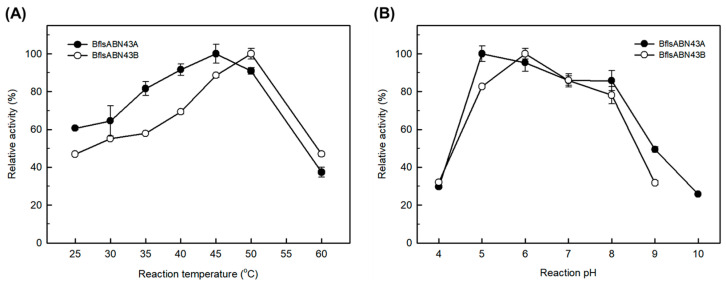
Effects of (**A**) temperature and (**B**) pH on the enzyme activities of endo-BflsABN43A and BflsABN43B. Relative activity on 0.5% debranched arabinan was determined under each reaction condition by using DNS-reducing sugar assay. Sodium acetate (pH 4~6), sodium phosphate (pH 7~8), Tris-HCl (pH 9), and Borate-NaOH (pH 10) buffers were used for enzyme activity assay.

The enzymatic activities of BflsABNs GH43 on debranched arabinan were determined using DNS-reducing sugar assay. BflsABN43A showed the highest activity (51.2 DU/mg) in 50 mM sodium acetate buffer (pH 5.0) at 45 °C. Meanwhile, BflsABN43B exhibited the highest activity (63.3 DU/mg) in 50 mM sodium acetate buffer (pH 6.0) at 50 °C. These hydrolases have a wide optimal pH range from 5.0 to 8.0, but their activities significantly decreased at pH 4.0 and 9.0 (Figure 5).

Most known microbial ABNs have their reaction optima at pH 5.0~8.0 and 40~60 °C. AbnA and Abn2 from *Bacillus subtilis*, for instance, are most active at 50~60 °C and pH 8.0 [43,44]. AbnB from *Geobacillus stearothermophilus* showed the highest activity at 60 °C and pH 5.0 [37]. Certain ABNs are exceptionally thermostable, including those from *Bacilus thermodenitrificans* [35], *Caldicellulosiruptor saccharolyticus* [36], and *Thermotoga petrophila* [39]. These thermostable ABNs were characterized by their optimal reaction temperatures at 70~75 °C, indicating a significant potential for industrial applications that require high-temperature processes.

**Figure 6:**
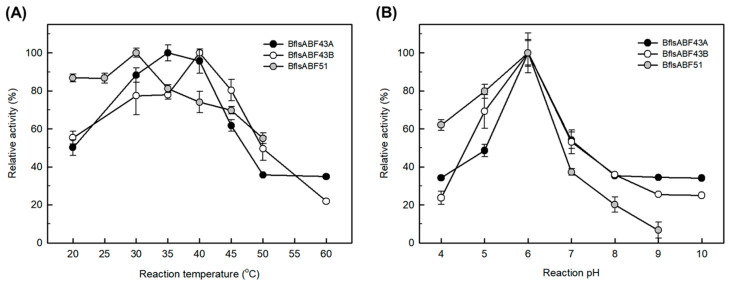
Effects of (**A**) temperature and (**B**) pH on the enzyme activities of exo-BflsABF43A, BflsABF43B, and BflsABF51. Relative activities on 0.5% sugar beet arabinan for ABF43A, debranched arabinan for ABF43B, and arabinobiose for ABF51 were determined under each reaction condition by using DNS-reducing sugar assay and l-arabinose assay, respectively. Sodium acetate (pH 4~6), sodium phosphate (pH 7~8), and Borate-NaOH (pH 9~10) buffers were used for enzyme activity assay.

Enzymatic activities of BflsABFs on arabinans and arabinobiose (A2) were also assessed by DNS-reducing sugar assay and l-arabinose assay methods, respectively. BflsABF43A showed the highest debranching activity on sugar beet arabinan (20.9 SU/mg) in 50 mM sodium acetate buffer (pH 6.0) at 35 °C. BflsABF43B exhibited the highest activity on debranched arabinan (61.3 DU/mg) at pH 6.0 and 40 °C. Both ABNs demonstrated a very narrow optimal pH around 6.0, with their activities significantly decreasing at pH 5.0 and 7.0. Meanwhile, BflsABF51 showed the highest activity (2.4 A2U/mg) at pH 6.0 and 30 °C. Like the other ABFs GH43, its activity was also rapidly decreased at pH 7.0 (Figure 6).

Enzymatic activity of ABFs from various microbes typically falls within the range of pH 5.0 to 8.0 and 40 °C to 60 °C, as reported from previous studies [16]. AbfA and Abf2 from *B. subtilis*, for instance, were reported to exhibit optimal activity at 50~60 °C and pH 8.0 [21]. Similarly, GAF from *Geobacillus* sp. showed the highest activity at 60 °C and pH 5.0 [13]. Highly thermostable ABFs have been identified in *Thermotoga maritima* [19] and *Caldicellulosiruptor saccharolyticus* [23], demonstrating the wide range of activity and stability profiles among microbial ABF enzymes.

To verify the substrate specificity of each arabinan hydrolase, the hydrolytic activity against various substrates was measured and compared with each other. Both endo-type hydrolases, BflasABN43A and ABN43B, exhibited much higher activity against debranched arabinan than against sugar beet arabinan. These ABNs GH43 exhibited much lower hydrolytic activity against linear arabino-oligosaccharides (LAOS), such as A2 with low degrees of polymerization (DPs), demonstrating typical characteristics of endo-hydrolases. However, intracellular BflsABN43B showed detectable hydrolytic activity (9.4 A3U/mg) even against short-chain LAOS like arabinotriose (A3), distinguishing it from extracellular BflsABN43A (Table 1).

As previously suggested, BflsABN43A, an extracellular endo-hydrolase, is predicted to play a crucial role in degrading arabinan polymers into appropriately sized AOS. Within the bifidobacterial cell, on the other hand, BflsABN43B is able to degrade the transported AOS into the shorter substrates, which can facilitate the successive saccharifying actions of intracellular exo-type ABFs by degrading transported AOS into the shorter substrates.

In the case of exo-BflsABFs, BflsABF43A exhibited hydrolytic activity only against branched substrates and showed higher activity towards branched arabino-oligosaccharides (BAOS) than sugar beet arabinan polymer. This hydrolase is predicted to be a debranching enzyme that hardly hydrolyzes linear substrates such as debranched arabinan and LAOS. In contrast, it was found that BflsABF43B is an exo-ABF that possesses high activity only against linear substrates, specifically acting on α-1,5-linkages. Despite being an exo-type ABF, this hydrolase also showed significant activity against debranched arabinan polymers, about 13% compared to that against LAOS. Meanwhile, BflsABF51 was almost unable to degrade the polymeric arabinan substrates, and its activity towards oligosaccharide substrates was also very low. However, unlike the other two ABFs, it was found to be active towards both LAOS and BAOS. These three types of intracellular ABF enzymes are expected to play synergistic roles in an efficient l-arabinose production. For example, BflsABF43A can facilitate the saccharifying action of BflsABF43B by debranching BAOS, whereas BflsABF51 with dual activities, despite its much low hydrolytic activity, might be capable of fulfilling the roles of both ABFs.

### 2.4. Hydrolytic Modes of Action of Arabinan Hydrolases

#### 2.4.1. endo-α-1,5-l-Arabinanases (BflsABNs)

**Figure 7:**
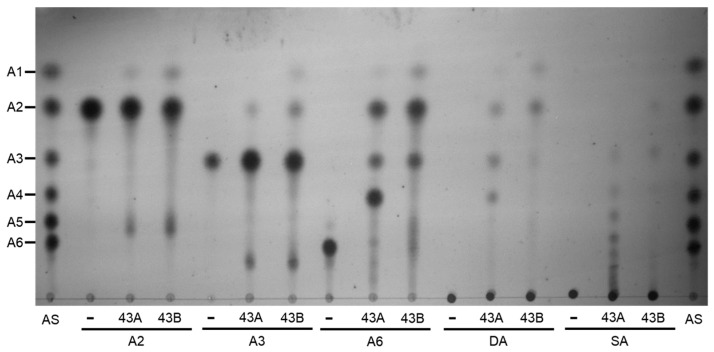
TLC analysis of the reaction products from various substrates hydrolyzed by endo-BflsABN43A and ABN43B. A total of 0.5% of substrate was reacted with each BflsABN43 under its optimal condition. 43A, BflsABN43A; 43B, BflsABN43B; AS, AOS standard from A1 (l-arabinose) to A6 (arabinohexaose); DA, debranched (linear) arabinan; SA, sugar beet (branched) arabinan. Symbol [-] indicates the reaction without enzyme.

To investigate the detailed hydrolytic modes of action for BflsABNs, each enzyme was reacted with 0.5% substrate, and the resulting hydrolysates were analyzed by TLC analysis. Although there were significant differences in degree, both enzymes showed very low hydrolytic activities against the short-chain AOS such as A2 and A3. However, with arabinohexaose (A6) as a longer substrate, both ABNs demonstrated detectable hydrolytic activities to produce A2 and arabinotetraose (A4) at the initial reaction step. As BflsABN43B possesses relatively higher enzyme activity on A4 than BflsABN43A, A2 was gradually accumulated as a major final product. Meanwhile, BflsABN43A could hydrolyze sugar beet arabinan to produce a series of BAOS, whereas BflsABN43B showed relatively low activity on sugar beet arabinan. When debranched arabinan was given as a substrate, it was confirmed that BflsABN43A produces A2, A3, and A4 as the main products, while BflsABN43B further hydrolyzed them to generate A2 as a finally accumulated product (Figure 7). The TLC results for enzymatic reaction products from each substrate were consistent with the substrate specificity for each enzyme shown in Table 1, allowing for an understanding of the hydrolytic modes of action for each arabinan-degrading enzyme.

#### 2.4.2. α-l-Arabinofuranosidases (BflsABFs)

**Figure 8:**
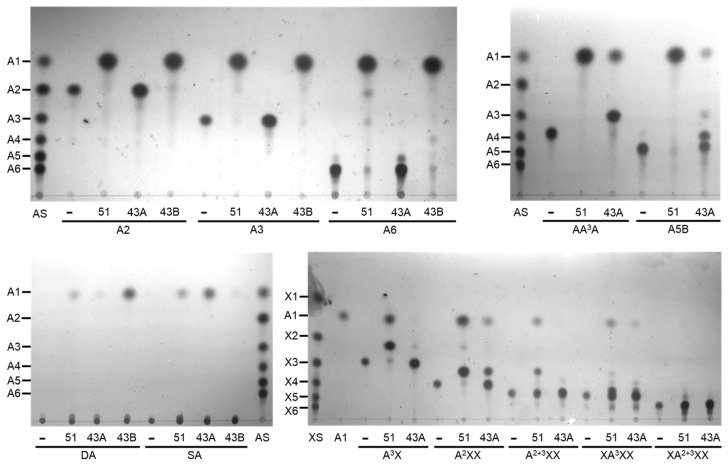
TLC analysis of the reaction products from various substrates hydrolyzed by exo-BflsABFs. A total of 0.5% of substrate was reacted with each BflsABF under its optimal condition. 51, BflsABF51; 43A, BflsABF43A; 43B, BflsABF43B; AS, AOS standard from A1 (l-arabinose) to A6 (arabinohexaose); XS, XOS standard from X1 (D-xylose) to X6 (xylohexaose); AA3A, 3^2^-α-l-arabinofuranosyl-arabinotriose; A5B, the mixture of AAA^3^A (3^2^-α-L-arabinofuranosyl-arabinotetraose) and AA^2+3^A (2^2^,3^2^-α-l-arabinofuranosyl-arabinotriose); DA, debranched (linear) arabinan; SA, sugar beet (branched) arabinan; A^3^X, 3^2^-α-l-arabinofuranosyl-xylobiose; A^2^XX, 2^3^-α-l-arabinofuranosyl-xylotriose; A^2+3^XX, 2^3^,3^3^-α-l-arabinofuranosyl-xylotriose; XA^3^XX, 3^3^-α-l-arabinofuranosyl-xylotetraose; XA^2+3^XX, 2^3^,3^3^-α-l-arabinofuranosyl-xylotetraose. Symbol [-] indicates the reaction without enzyme.

BflsABF43A specifically hydrolyzed α-1,2- and/or α-1,3-arabinofuranosyl branches in BAOS and sugar beet arabinan to produce LAOS and debranched arabinan, respectively, while it could hardly cleave α-1,5-linked backbones in substrates. Extremely low activity towards α-1,5-linkages can explain the resistance to the hydrolysis of linear substrates. To understand the detailed modes of debranching action, BAOS and AXOS were reacted with BflsABF43A, and the resulting hydrolysates were identified by TLC (Figure 8).

In the case of AA^3^A as a substrate, BflsABF43A first attacked and removed α-1,3-linked branch to produce linear A3 and l-arabinose. Against a BAOS mixture of AAA^3^A and AA^2+3^A, the α-1,3-linked branch of AAA^3^A was rapidly removed by debranching activity of BflsABF43A at the early reaction stage. After the preferred debranching action of AAA^3^A, the residual AA^2+3^A could be degraded into A3 and l-arabinose at an extremely slow rate. Among the AXOS substrates, BflsABF43A hydrolyzed A^2^XX into xylotriose and l-arabinose very slowly, while it could hardly remove l-arabinofuranosyl branches in the other AXOS, such as A^3^X, A^2+3^XX, XA^3^XX, and XA^2+3^XX. As a result, BflsABF43A is an arabinan-specific exo-hydrolase with high debranching activity towards α-1,2- and/or α-1,3-linked BAOS and sugar beet arabinan, generating various linear products. Meanwhile, BflsABF43B could specifically hydrolyze α-1,5-linked l-arabinofuranosyl backbone in linear forms of substrates, such as LAOS and debranched arabinan, to release l-arabinose as a final product. It cannot attack sugar beet arabinan, a branched substrate. These results explain that BflsABF43B is an arabinan-specific exo-α-1,5-l-arabinofuranosidase.

BflsABF51 could completely hydrolyze BAOS into only l-arabinose, which revealed that it can hydrolyze all α-1,2-, α-1,3-, and α-1,5-l-arabinofuranosyl linkages in only short-chain AOS. Unlike BflsABFs GH43, as shown in Table 1, however, BflsABF51 could not hydrolyze the arabinan polymers, and its activity on AOS was much lower than that of BflsABFs GH43. Instead, it preferentially attacked α-1,2- and/or α-1,3-arabinofuranosyl linkages at the non-reducing ends in AXOS. As a result, BflsABF51 is an exo-acting ABF with specific debranching activities on oligomeric substrates such as BAOS and AXOS, as well as hydrolyzing α-1,5-linkages in AOS.

## 3. Discussion

Microbial arabinan utilization systems are composed of (1) various exo- and endo-acting hydrolases for arabinan degradation into l-arabinose, (2) membrane-associated transporter proteins for arabinan and/or its hydrolysates taken into the cell, and (3) a series of enzymes involved in l-arabinose metabolism. Gene clusters for arabinan utilization have been discovered in the genomes of *Bacillus subtilis* [21,43,44], *Hypocrea jecorina* [45], *Corynebacterium glutamicum* [46], *Geobacillus stearothermophilus* [47], *Caldanaerobius polysaccharolyticus* [41], and *Lactobacillus crispatus* [42].

**Figure 9:**
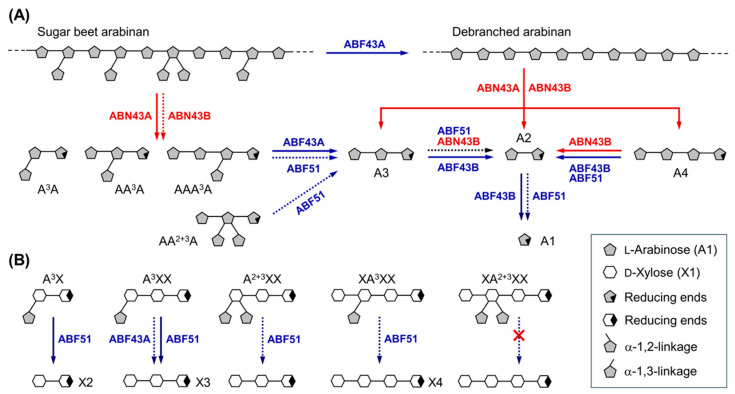
Synergistic modes of action for endo-BflsABNs and exo-BflsABFs on (**A**) arabinans and (**B**) arabinoxylo-oligosaccharides. Solid and dashed arrows denote fast and slow steps for enzymatic degradation of each substrate, respectively.

From the genome of *Bf. longum* subsp. *suis* ATCC 27533, a total of five genes encoding core arabinan-degrading enzymes were functionally expressed, and their enzymatic properties were characterized in this study. The corresponding gene cluster contains two endo-ABNs GH43 (BflsABN43A and ABN43B), two exo-GH43 ABFs (BflsABF43A and ABF43B), and an exo-ABF GH51 (BflsABF51), as well as an *ara* operon for l-arabinose metabolism. Comparative enzymatic characterization proposed that these hydrolases possess their own unique modes of hydrolytic action, which can synergistically work together for the efficient degradation of arabinan into l-arabinose. At first, the extracellular BflsABN43A is expressed and secreted into the medium containing AOS and arabinans. BflsABN43A can hydrolyze sugar beet arabinan into a series of BAOS as the major hydrolyzed products, while it can also degrade debranched arabinan to produce LAOS such as A2, A3, and A4 with small amount of l-arabinose. The resulting AOS and BAOS are uptaken through the transporters integrated in the cell membrane, and further hydrolyzed via the concerted actions of intracellular exo- and/or endo-hydrolases, including BflsABF43A, BflsABF43B, BflsABF51, and BflsABN43B. Within the cell, BAOS can be converted into linear AOS by the debranching action of BflsABF43A, and the resulting LAOS can be shortened by the endo-action of intracellular BflsABN43B. Short-chain AOS will be completely saccharified into l-arabinose via the exo-actions of BflsABF43B. The detailed hydrolytic modes of action for these endo-and exo-type arabinan-degrading enzymes from *Bf. longum suis* and their predictive synergism are schematically proposed (Figure 9).

l-arabinose in the cytoplasm is moved on the metabolism being coupled with the pentose phosphate pathway. In contig-18, it was found that the putative *ara* operon genes consist of *araA*, *araD*, and *araB*, encoding l-arabinose isomerase, l-ribulose-5-phosphate 4-epimerase, and l-ribulose kinase, respectively. These enzymes are involved in the metabolic pathway that converts l-arabinose into d-xylulose-5-phosphate. Accordingly, it was expected that *Bf. longum suis* can utilize l-arabinose as an energy source. Comparative genomics revealed the gene regulation of bifidobacterial l-arabinose and AOS utilization system [58]. The AauR regulator was reported as a LacI family transcriptional factor in various bifidobacteria. Several arabinan-degrading enzyme genes and ABC transporter genes were located near the AauR transcriptional regulator gene.

The homologs to BflsABN43A have been found in various bifidobacteria, such as *Bf. longum*, *Bf. asteroids*, *Bf. pseudolongum*, and *Bf. longum* subsp. *infantis*. Among them, *Bf. longum* NCC2705 and *Bf. longum* subsp. *infantis* CECT7210 were chosen for the comparative analyses of arabinan utilization gene cluster with *Bf. longum suis* ATCC 27533. These three *Bifidobacterium* species share highly similar structure of gene clusters for arabinan utilization with each other. In conclusion, large numbers of *Bf. longum* spp. are expected to possess *ara* operon and/or various arabinan-degrading enzyme genes in their own genomes. In order to understand the details of arabinan utilization gene clusters and their cooperative functions in bifidobacteria, further studies on the transcriptional regulations and transporter systems for AOS and arabinans, as well as the functional characterization of arabinan hydrolases.

## 4. Materials and Methods

### 4.1. Microorganisms and Plasmids

*Escherichia coli* MC1061 and BL21(DE3) were used as the host strains for gene cloning and expression. Each *E. coli* transformant was cultured in Luria–Bertani (LB) medium (1% tryptone, 1% sodium chloride, 0.5% yeast extract) containing 100 μg/mL of ampicillin. *Bifidobacterium longum* subsp. *suis* ATCC 27533 (= DSM 20211 = KCTC 3229) was obtained from Korean Collection for Type Cultures (KCTC, Daejeon, Republic of Korea). The plasmid vectors, pHCXHD [59] and pET-21a(+) (Novagen, Bagsværd, Denmark), were utilized for constitutive and IPTG-inducible gene expressions, respectively.

### 4.2. Enzymes and Chemical Reagents

Restriction enzymes and Pyrobest DNA polymerase were provided from Takara Biomedical (Otsu, Japan) and New England Biolabs (Beverly, MA, USA). Arabinans, various oligosaccharides (AOS, A5B, XOS, and AXOS), and an l-Arabinose/d-Galactose assay kit was obtained from Megazyme International (Wicklow, Ireland). Other chemical reagents used in this study were purchased from Sigma-Aldrich (St. Louis, MO, USA), and Merck (Darmstadt, Germany). Oligonucleotide primers were synthesized by Bioneer (Daejeon, Republic of Korea) and DNA sequencing analyses were performed by SolGent (Daejeon, Republic of Korea).

### 4.3. Gene Cloning of Arabinan Hydrolases

Genomic DNA template was isolated from *Bf*. *longum suis* using a DNeasy Blood & Tissue kit (Qiagen, Hilden, Germany). Five sets of primers, BflsAN43AΔS-F (5′-AAAGGAGATATCATATGTCGTCGAACGCCAAGAAC-3′) and BflsAN43A-R (5′-GGTGATGGTGCTCGAGCTTCTGCGAGGCCCAG-3′), BflsAN43B-F (5′-AAGAAGGAGATATACATATGACCGCAACCACCTC-3′) and BflsAN43B-R (5′-GGTGGTGGTGGTGCTCGAGGCCCTCGATGCGGGC-3′), BflsAF43A-F (5′-GAAAAAGGAGATATCATATGTCCGACTCCTACTCA-3′) and BflsAF43A-R (5′-GGTAGTGATGGTGCTCGAGCCGCACGGCGATTTCC-3′), BflsAF43B-F (5′-GAAAAAGGAGATATCATATGACTGTTTACAACAATCC-3′) and BflsAF43B-R (5′-GGTAGTGATGGTGCTCGAGTGCCTCTGCGGCCAGG-3′), BflsAF 51-F (5′-GAAAAAGGAGATATCATATGGCAGACAAACTGGTAG-3′), and BflsAF51-R (5′-GGTAGTGATGGTGCTCGAGGCCTCGTACGGCAAGC-3′), were applied to PCR amplification by using Pyrobest polymerase (Takara Biomedical) and thermal cycler C-1000 (Bio-Rad, Hercules, CA, USA) with the following steps: an initial denaturation at 98 °C for 1 min followed by 30 repeated cycles at 98 °C for 30 s, 50 °C for 30 s, 72 °C for 3 min, and a final cycle at 72 °C for 10 min. PCR products were purified by using *Accu*Prep Gel Purification Kit (Bioneer), which were cleaved with *Nde*I/*Xho*I and cloned into the expression vectors, pHCXHD or pET-21a(+). The resulting plasmids were designated as pHBflsAN43A, pEBflsAN43B, pHBflsAF43A, pHBflsAF43B, and pEBflsAF51, respectively. The recombinant plasmid was purified using *Accu*Prep Mini Plasmid Extraction Kit (Bioneer) according to the protocol from the supplier.

### 4.4. Gene Expression and Enzyme Purification

Each recombinant plasmid was transformed into *E. coli* MC1061 or BL21(DE3) as the host strains for constitutive and inducible gene expression, respectively, by using calcium chloride method. *E. coli* transformant was cultured in LB broth with final 0.1 mM IPTG and 100 μg/mL ampicillin at 37 °C for 15 h. The grown cell was disrupted by ultrasonicator VCX750 (Sonics & Materials, Newtown, CT, USA). Recombinant enzyme with C-terminal six-histidines was purified via HisTrap-FF column chromatography by using AKTA Prime (GE Healthcare, Uppsala, Sweden). Gene expression level and enzyme purity were confirmed by using 12% SDS-PAGE. Protein molecular weight markers for SDS-PAGE were purchased from Bio-Rad (Hercules, CA, USA). The protein concentration was determined by a BCA protein assay kit (Pierce Biotechnology, Rockford, IL, USA) with a microplate reader (BIO-TEK, Winooski, VT, USA) according to the instruction from the supplier.

### 4.5. Enzyme Activity Assays

#### 4.5.1. DNS (3,5-Dinitrosalicylic Acid) Reducing Sugar Assay

The amount of reducing sugar was measured by the 3,5-dinitrosalicylic acid (DNS) method. The enzyme reaction mixture was composed of 50 μL of 5% (*w*/*v*) debranched arabinan and sugar beet arabinan in optimal reaction buffer for each enzyme, 10 μL of reaction buffer, and 10 μL diluted enzyme solution. Reaction mixture without enzyme solution in tube was incubated at reaction temperature for 3 min, and then diluted enzyme solution was added and incubated for 10 min. The reaction was stopped by adding 300 μL of DNS solution (3,5-dinitrosalicylic acid 10.6 g, Na, K-tartrate 306 g, NaOH 19.8 g, Na-metabisulfite 8.3 g, phenol 7.6 mL, and water 1416 mL). The reaction mixture was boiled for 5 min and cooled in ice-cold water. Absorbance of the reaction mixture (300 μL) was measured at 550 nm using a microplate reader (BIO-TEK). One unit of enzyme activity was defined as the amount of enzyme used for the production of 1 μmol of l-arabinose equivalent per min under optimal reaction conditions.

#### 4.5.2. l-Arabinose Assay

l-arabinose concentration in the reaction mixture was determined at 340 nm using an l-Arabinose/d-Galactose Assay Kit (K-ARGA, Megazyme), according to the protocol from the supplier. One unit of enzyme activity was defined as the amount of enzyme used for the production of 1 μmol of l-arabinose per min under optimal reaction conditions.

### 4.6. Thin-Layer Chromatography (TLC) Analysis

A Merck 60F_254_ silica gel plate was pre-activated by placing it for 30 min at 80 °C. Each sample was spotted on a silica plate and separated in a TLC chamber containing a solvent of chloroform/acetic acid/water (6:7:1, *v*/*v*/*v*). The plate was visualized by dipping into a methanol solution containing 0.3% (*w*/*v*) *N*-(1-naphthyl)-ethylendiamine and 5% (*v*/*v*) H_2_SO_4_, and being dried for 10 min at 120 °C.

### 4.7. Three-Dimensional Structure Modeling

Prediction and modeling of enzyme structure was performed by using AlphaFold2 [https://alphafold.ebi.ac.uk] available on the web server. Each enzyme structure was visualized by using a molecular graphics tool, PyMOL 1.5.0.3 [http://pymol.org].

## 5. Conclusions

Arabinan, a type of hemicellulose polymer, is broken down into l-arabinose through the cooperative actions of various endo- and exo-type hydrolases. In this study, it was found that the diverse hydrolase genes were clustered in the genome of *Bifidobacterium longum suis*, and the ORFs encoding two endo-ABNs and three exo-ABFs were individually expressed in *E. coli*, elucidating their functional roles in arabinan hydrolysis. These enzymes are expected to act synergistically on arabinan, enhancing the substrate accessibility and hydrolytic activity of each other. The produced l-arabinose can be utilized as a carbon source, potentially exhibiting a prebiotic effect by stimulating the growth of bifidobacteria.

## Figures and Tables

**Table 1:** Substrate specificities of endo- and exo-arabinan hydrolases from *Bf. longum suis*.

Substrate ^1^	Specific Activity (U/mg) ^2^				
	BflsABN43A	BflsABN43B	BflsABF43A	BflsABF43B	BflsABF51
Arabinobiose (A2)	NA	NA	NA	439.65 ± 16.09	1.21 ± 0.08
Arabinotriose (A3)	NA	9.38 ± 1.08	NA	444.97 ± 21.46	0.47 ± 0.03
BAOS (AA^3^A)	NA	NA	46.00 ± 1.29	NA	2.16 ± 0.18
Debranched arabinan (DA)	51.19 ± 0.97	63.27 ± 1.86	NA	61.34 ± 0.63	NA
Sugar beet arabinan (SA)	9.85 ± 0.32	2.77 ± 0.06	20.87 ± 1.08	NA	NA

^1^ Abbreviations: BAOS, branched arabinooligosaccharides; AA^3^A, 3^2^-α-l-arabinofuranosyl arabinotriose; NA, no (detectable) activity. ^2^ Enzyme activities on oligomeric and polymeric substrates were determined by using l-arabinose assay and DNS-reducing sugar assay, respectively.

## Data Availability

The data presented in this study are available on request from the corresponding author.
